# Macrocytosis during sunitinib treatment predicts progression-free survival in patients with metastatic renal cell carcinoma

**DOI:** 10.1007/s12032-016-0818-9

**Published:** 2016-08-30

**Authors:** Jakub Kucharz, Agnieszka Giza, Paulina Dumnicka, Marek Kuzniewski, Beata Kusnierz-Cabala, Pawel Bryniarski, Roma Herman, Aneta Lidia Zygulska, Krzysztof Krzemieniecki

**Affiliations:** 1 Department of Experimental and Clinical Surgery, Jagiellonian University Medical College, Michalowskiego 12, 31-126 Kraków, Poland; 2 Department of Hematology, Jagiellonian University Medical College, Kopernika 17, 31-501 Kraków, Poland; 3 Department of Medical Diagnostics, Jagiellonian University Medical College, Medyczna 9, 30-688 Kraków, Poland; 4 Department of Nephrology, Jagiellonian University Medical College, Kopernika 15, 31-501 Kraków, Poland; 5 Department of Diagnostics, Chair of Clinical Biochemistry, Jagiellonian University Medical College, Kopernika 15A, 31-501 Kraków, Poland; 6 Students Scientific Society, Jagiellonian University Medical College, Sw. Anny 12, 31-008 Kraków, Poland; 7 Department of Pediatrics, Gastroenterology and Nutrition, Jagiellonian University Medical College, Wielicka 265, 30-663 Kraków, Poland; 8 Department of Oncology, University Hospital in Krakow, Sniadeckich 10, 31-531 Kraków, Poland; 9 Department of Oncology, Jagiellonian University Medical College, Sniadeckich 10, 31-531 Kraków, Poland

**Keywords:** Metastatic renal cell carcinoma (mRCC), Predictive factors, Macrocytosis, MCV, Progression-free survival (PFS)

## Abstract

Sunitinib, a multi-targeted receptor tyrosine kinase inhibitor, is a first-line treatment for metastatic renal cell carcinoma (mRCC) in patients in ‘low’ and ‘intermediate’ Memorial Sloan Kettering Cancer Center and Heng risk groups. Disruptions of hematopoiesis, such as anemia, neutropenia, and thrombocytopenia, are typically observed during sunitinib treatment. When it comes to RBC parameters, an increase in mean cell volume (MCV) tends to occur, meeting the criteria for macrocytosis in some patients (MCV > 100 fL). We examined changes in RBC parameters of 27 mRCC patients treated with sunitinib (initial dose of 50 mg/day, 6-week treatment: 4 weeks on, 2 weeks off) and correlated them with progression-free survival time (PFS). Patients who had macrocytosis after 3 treatment cycles had significantly longer PFS than those whose MCV stayed less than 100 fL (not reached vs. 11.2 months, *p* < 0.001). We also found a correlation between MCV values after the first and third treatment cycles and the risk of progression: HR of 0.9 (0.81–0.99) and 0.76 (0.65–0.90) per 1 fL increase in MCV, respectively. The mechanism of MCV elevation during sunitinib treatment has not yet been fully explained. One of the probable causes is sunitinib’s inhibitory influence on c-Kit kinase, as is the case with imatinib. For mRCC patients, this phenomenon could help predict PFS, but since our sample was small, further studies are essential.

## Introduction

Sunitinib is a TKI that acts on various receptors and kinases, including the vascular endothelial growth factor receptors 1–3 (VEGFR1–VEGFR3), the platelet-derived growth factor receptors (PDGFRα and PDGFRβ), the stem cell factor receptor (Kit), the Fms-like tyrosine kinase 3 (Flt-3), and the colony-stimulating factor-1 receptor (CSF-1R) [1]. The medical community typically recommends sunitinib as a first-line treatment in mRCC patients classified as belonging to ‘low’ and ‘intermediate’ risk group according to MSKCC and Heng criteria [2].

However, sunitinib is associated with adverse events that were previously uncommon in systemic treatment. These include cutaneous, vascular, and mucosal toxicities, such as hand–foot syndrome, skin rash, and hypertension, and endocrine toxicities like hypothyroidism [3, 4]. Moreover, abnormalities in laboratory tests, including cytopenias (leukopenia, neutropenia, anemia, and thrombocytopenia), and disruptions of renal and liver function, are also observed during sunitinib treatment [3–6].

These adverse effects seem to be related to sunitinib mechanisms of action, i.e., its inhibition of signaling pathways.

In a phase III clinical trial, Motzer et al. [4] found that treating mRCC patients with sunitinib resulted in significantly higher response rates (31 vs. 6 %) and PFS (11.0 vs. 5.0 months) than treatment with interferon alpha, but clinical practice shows significant differences in both treatment outcomes and sunitinib toxicity across patients. Studies have shown that sunitinib-induced adverse effects like hypertension, hypothyroidism, hand–foot syndrome (HFS), myelosuppression, and hypothyroidism (as well as their co-occurrence) could signal the drug’s activity in the body and help predict treatment outcome parameters like ORR, PFS, and OS [7–10].

A number of studies that evaluated complete blood count (CBC) during sunitinib treatment found red blood cell (RBC) disruptions. In addition to lower RBC and HGB counts, authors have noted increases in MCV, which were reversible after treatment completion [11]. The mechanism that causes this phenomenon has not yet been fully explained.

Our aim in this study was to assess whether erythrocyte indices, especially macrocytosis, predicted PFS in a homogenous group of clear cell mRCC patients.

## Materials and methods

### Patient selection and therapeutic procedure

This was a retrospective study of 27 patients treated at the Department of Oncology at the University Hospital in Krakow, Poland, between 2008 and 2013, and selected according to the following inclusion criteria as follows: diagnosis of clear cell mRCC, application of sunitinib as a first-line treatment for the mRCC, prior nephrectomy (total or nephron-sparing surgery), and good or intermediate MSKCC risk prognosis. All patients received sunitinib on a standard schedule (initial dose of 50 mg/day, 6-week course: 4 weeks on, 2 weeks off). The hematology test profile included total erythrocyte count (RBC), hemoglobin (HGB), hematocrit (HCT), and erythrocyte indices: mean cell volume (MCV), mean cell hemoglobin (MCH), mean cell hemoglobin concentration (MCHC), and red cell distribution width—coefficient of variation (RDW-CV). We received permission to conduct this study from the Jagiellonian University Bioethics Committee (permission number KBET/45/B/2013).

### Data collection and evaluation criteria

Our data set consisted of patient demographics, laboratory test results (including CBC, TSH, cobalamine, and folic acid levels), treatment delays, treatment duration, and treatment outcomes. The CBC profile was evaluated before starting sunitinib (baseline values) and at the end of each course of treatment. TSH was evaluated after every other course of treatment, while cobalamine and folic acid levels were assessed whenever the supervising physician prescribed it. Hematology parameters were measured using the 5 diff Sysmex XE 2100 Hematological Analyzer (Sysmex Corp., Japan). Vitamin B12, folic acid, and TSH levels were measured with electrochemiluminescentric ECLIA methodology on Cobas 6000 (Roche Diagnostic, Mannheim, Germany). The reference range was 141–489 pmol/L (191–663 pg/mL) for serum vitamin B12; 10.4–42.4 nmol/L (4.6–18.7 ng/mL) for folic acid; and 0.27–4.20 μIU/mL for TSH. Laboratory tests were carried out by the Diagnostics Department of the University Hospital in Krakow.

### Statistical analysis

Nominal variables are summarized as the number of patients (percentage of the group) and continuous variables as median (lower/upper quartile) or mean ± SD, according to distribution (tested for normality using Shapiro–Wilk test). Baseline RBC and erythrocyte indices were compared with values after 3 and 5 cycles, using repeated-measures ANOVA with the Bonferroni post hoc test. Differences between groups with and without hypothyroidism as well as with or without vitamin B12 deficiency were tested with a *t* test. PFS values were calculated with a start date at the beginning of the sunitinib treatment and end date at progression or death, or censored at the end of the study, in case of patients who remained in treatment thereafter. PFS was estimated using the Kaplan–Meier method and compared with the log-rank test. The associations between RBC and erythrocyte indices with PFS were studied using univariate and MSKCC-adjusted Cox proportional hazards models; resulting hazard ratios (HRs) are reported with 95 % confidence intervals.

## Results

Clinical characteristics of the study group (*N* = 27) at the start of the sunitinib treatment are shown in Table 1. During the study period, we observed progression in 20 patients (74 %), after a minimum of 2 cycles (i.e., 12 weeks) and a maximum of 21 cycles (i.e., about 29 months). The remaining 7 patients (26 %) continued treatment at the end of the study. Twenty-four patients stayed in treatment after 3 cycles, and 20 patients were in treatment for at least 6 cycles (>8 months). Median PFS for the whole study group was 12.5 months (lower/upper quartile 5.2/29.4 months).

**Table 1:** Baseline characteristics of patients

	Clear cell mRCC patients (*N* = 27)
Age, years	65 (59/69)
Male sex, *N* (%)	18 (67)
*Fuhrman*	
Grade 1–2, *N* (%)	10 (37)
Grade 3–4, *N* (%)	17 (63)
Nephrectomy (total or nephron sparing), *N* (%)	27 (100)
Time from diagnosis to systemic treatment <1 year, *N* (%)	14 (52)
*ECOG performance score*	
0, *N* (%)	13 (48)
1, *N* (%)	13 (48)
2, *N* (%)	1 (4)
*MSKCC prognosis*	
Favorable, *N* (%)	8 (30)
Intermediate, *N* (%)	19 (70)
*Metastases*	
No metastases	5 (19)
Lung, *N* (%)	16 (59)
Liver, *N* (%)	11 (41)
Bone, *N* (%)	5 (19)
1 Site, *N* (%)	15 (56)
2 or more sites, *N* (%)	7 (26)

**Fig. 1:**
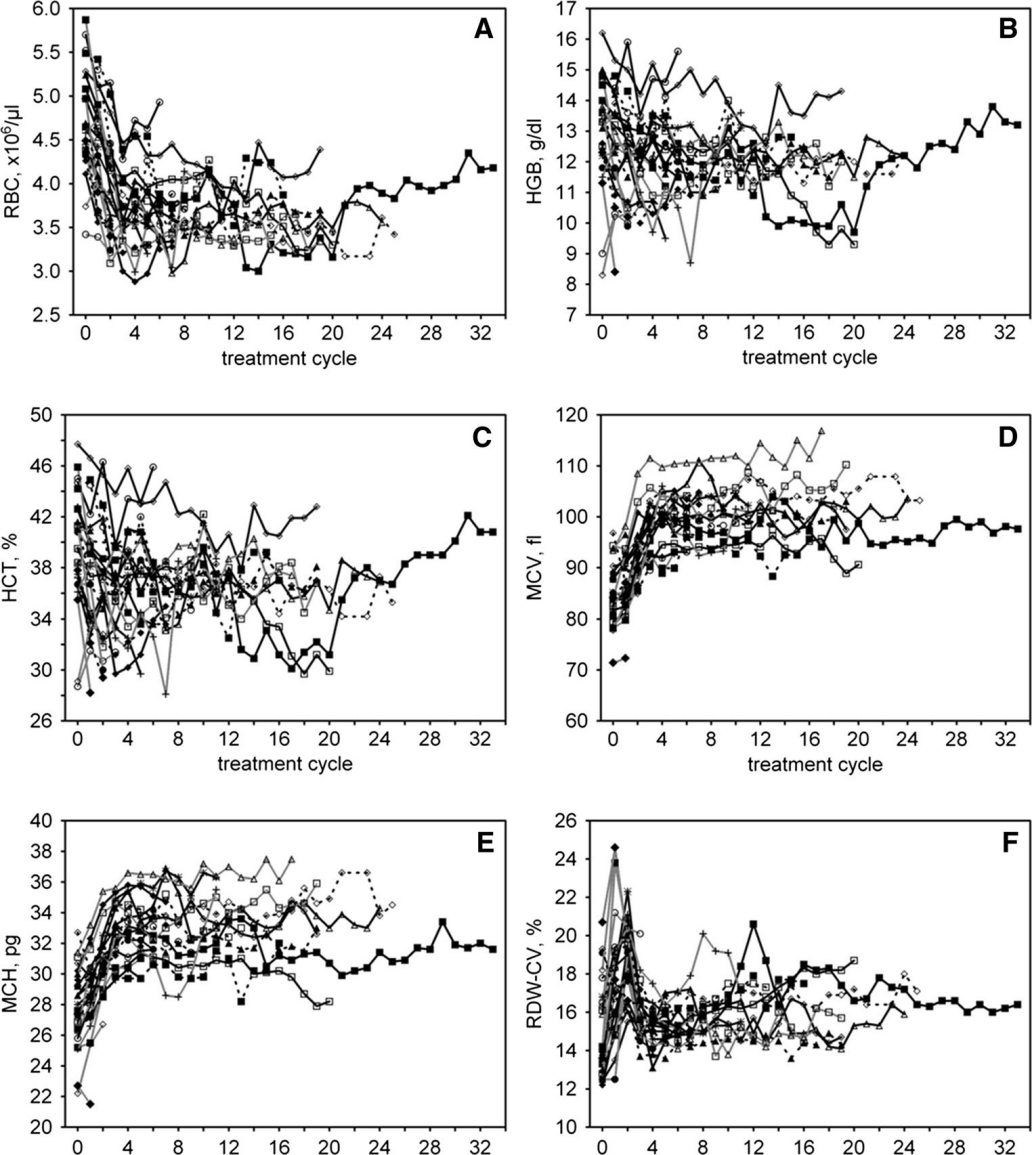
Case profiles showing changes in RBC counts and indices for the whole group of mRCC patients (*N* = 27) during sunitinib treatment. Treatment cycle 0 denotes baseline results. **a** RBC change; **b** HGB change; **c** HCT change; **d** MCV change; **e** MCH change; **f** RDW-CV change

We observed significant changes in RBC counts and other indices, and these were most pronounced during the first 3 treatment cycles (Fig. 1). After the first cycle, we observed lower RBC counts in 25 patients (93 %); after 3 cycles, all but one patient had RBC counts lower than baseline. After the first treatment cycle, MCV and MCH increased in 23 patients (85 %). After 3 cycles, all patients who stayed in treatment (*N* = 24) had MCH values higher than baseline, and the MCV values of all but one patient were also higher than baseline. We checked the statistical significance of these changes in the subgroup of 20 patients who were in treatment for at least 6 cycles (Table 2), and our analysis confirmed that the significant changes occurred during the first 3 treatment cycles, followed by a plateau. The simultaneous decrease in HGB concentrations and HCT was much less consistent (Fig. 1); however, average values after 3 treatment cycles were also significantly lower than baseline (Table 2). RDW-CV showed a fast initial increase, followed by a decrease and then a plateau (Fig. 1). Only MCHC did not show significant changes after starting sunitinib (Table 2, data not shown in Fig. 1).

**Table 2:** Changes in RBC counts and indices in patients who were in treatment for at least 6 cycles (*N* = 20)

	Baseline	After 3 cycles	After 5 cycles	*p*
RBC, ×10^6^/µl	4.86 ± 0.52	3.73 ± 0.39	3.74 ± 0.44	<0.001^a^
HGB, g/dl	13.6 ± 1.1	12.1 ± 1.0	12.3 ± 1.4	<0.001^a^
HCT, %	41.1 ± 3.3	36.4 ± 3.4	37.0 ± 3.8	<0.001^a^
MCV, fL	85.5 ± 5.6	98.0 ± 5.3	99.3 ± 5.1	<0.001^a^
MCH, pg	28.4 ± 2.2	32.7 ± 1.8	32.8 ± 1.9	<0.001^a^
MCHC, g/dl	33.1 ± 0.7	33.3 ± 1.0	33.1 ± 0.9	0.4
RDW-CV, %	14.5 ± 2.2	15.8 ± 1.1	15.3 ± 0.9	0.022^b^

^a^In post hoc comparisons, results after 3 and 5 cycles differ from baseline but not from each other

^b^Results after 3 cycles differ from baseline while other differences are insignificant

We monitored TSH concentrations throughout treatment, and therapy-induced hypothyroidism occurred in 12 patients (44 %). However, RBC counts and indices did not differ significantly between patients who developed hypothyroidism and those who did not. In particular, MCV values after 3 treatment cycles were similar for both groups (96.9 ± 5.3 vs. 98.4 ± 5.6 fL; *p* = 0.5). The concentrations of vitamin B12 and folic acid were available for only 8 patients (those with highest MCV): none had folic acid deficiency, but four patients had vitamin B12 levels below the reference range. Although patients with vitamin B12 deficiency had higher MCV (e.g., after 3 cycles: 104.9 ± 4.9 vs. 100.0 ± 0.6 fL), the differences were not statistically significant (*p* = 0.2).

**Fig. 2:**
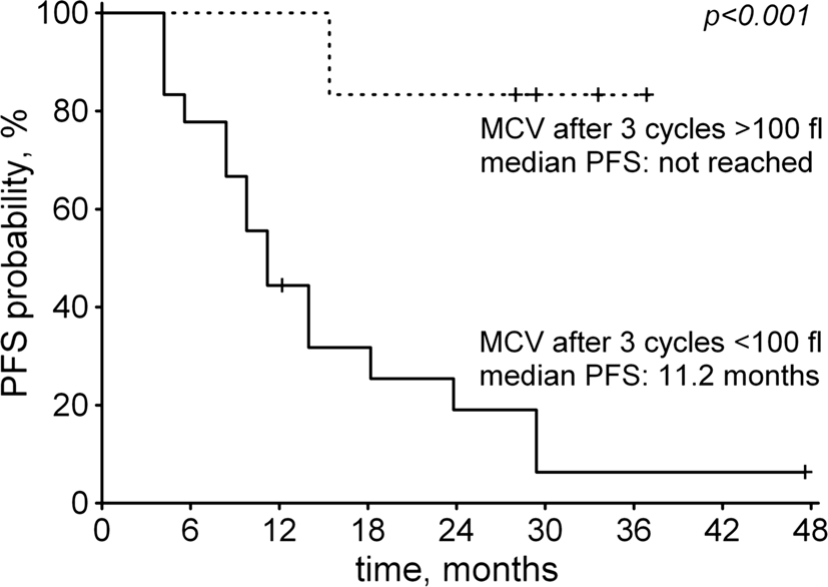
Kaplan–Meier curves showing progression-free survival in patients with apparent macrocytosis (MCV > 100 fL) after 3 cycles of sunitinib treatment (*dashed line*) and those with lower MCV values (*solid line*). Analysis included patients who were in treatment for at least 3 cycles (*N* = 24)

During sunitinib treatment, higher MCV and MCH values were consistently correlated with longer PFS times in both univariate and MSKCC-adjusted Cox regressions (Table 3). The correlations were observed as early as after the first treatment cycle and became even stronger after 3 cycles, at which point we also observed apparent macrocytosis (MCV > 100 fL) in 6 patients (25 %), who eventually had significantly longer PFS times, as shown in Fig. 2.

**Table 3:** Significant hazard ratios (95 % confidence intervals) for progression in MSKCC-adjusted Cox regression

	MCV, per 1 fL	MCH, per 1 pg
After 1 cycle (*N* = 27)	0.90 (0.81–0.99)	0.68 (0.53–0.86)
After 3 cycles (*N* = 24)	0.76 (0.65–0.90)	0.45 (0.28–0.73)
After 5 cycles (*N* = 20)	0.76 (0.64–0.90)	0.58 (0.38–0.90)

## Discussion

Current diagnostic standards during systemic treatment mandate the inclusion of automated CBC counts. When it comes to adverse effects of sunitinib therapy on the hematopoietic system, manual microscope examination of the peripheral blood smear is also justified. Such microscopic analysis allows for significantly earlier diagnosis of macrocytosis. In addition, in automated MCV analysis, falsely elevated MCV values can be caused by hyperglycemia, hyperleukocytosis, and cold agglutinins [12].

The macrocytosis is a blood condition in which red blood cells are larger than normal. Normal MCV value ranges from 80 to 100 fL [13]. Macrocytosis without associated anemia can also be a non-pathological phenomenon in newborns and pregnant women. When macrocytic anemia is diagnosed, it can be either megaloblastic or non-megaloblastic. Megaloblastic anemia is caused by vitamin B12 and folic acid deficiency, which can lead to ineffective, dysplastic hematopoiesis. Other causes of macrocytosis, which lead to non-megaloblastic anemia, include medications (e.g., anticonvulsants, and anti-bacterial and antiretroviral drugs), alcoholism, liver disease, hypothyroidism, myelodysplastic syndrome, and reticulocytosis (Table 4) [12, 14].

**Table 4:** Common causes of macrocytosis [14]

Megaloblastic macrocytosis (vitamin B12 and/or folate deficiency)	Non-megaloblastic macrocytosis	False elevation mean corpuscular volume (MCV)
1. Atrophic gastritis	1. Alcohol abuse	1. Cold agglutinin
2. Enteral malabsorption	2. Medication side effects	2. Hyperglycemia
3. Human immunodeficiency virus treatment	3. Myelodysplasia	3. Marked leukocytosis
4. Anticonvulsants	4. Hypothyroidism	
5. Primary bone marrow disorders	5. Liver disease	
6. Nitrous oxide abuse	6. Hemolysis	
7. Inherited disorders	7. Hemorrhage	
	8. Chronic obstructive pulmonary disease	
	9. Splenectomy	

Thus, the proper diagnostic differentiation of macrocytosis requires a detailed patient history, laboratory exploration of parameters that might be causing the macrocytosis, manual microscope evaluation of the peripheral blood smear, and automated laboratory blood tests that take the number of both reticulocytes and anisocytosis (RDW-CV) into account. Bone marrow analysis, the most invasive test, may be necessary in megaloblastosis, in order to rule out myelodysplastic syndrome. Treatments relying on tyrosine kinase inhibitors (TKIs), such as imatinib, sunitinib, and sorafenib, can now be counted among the possible causes of macrocytosis with previously unexplained pathomechanisms [11, 15].

In this study, we observed drops in RBC counts and MCV elevation after the first treatment cycle. The most significant RBC drop, along with increases in MCV and MCH values, took place during the first 3 to 4 treatment cycles, after which RBC, MCV, and MCH values stabilized. This finding diverges from Kloth et al. [16], who found elevated MCV only after approximately 3 months of sunitinib therapy. We should add that Kloth et al. analyzed MCV changes in a non-homogenous group of patients treated with sunitinib for various diseases and in various/successive lines of treatment.

We found that patients who developed macrocytosis after 3 sunitinib treatment cycles had longer PFS times than those without macrocytosis. Greatest changes in RBC parameters occurred after 126 days (3 treatment cycles), which corresponds to the life span of peripheral erythrocytes, which is approximately 120 days. However, increases in MCV values, noted as early as after the first treatment cycle, were significantly correlated with longer PFS.

Kloth et al. [16] explored the relationship between the development of macrocytosis and various survival parameters of patients (*n* = 533) treated with TKIs (including sunitinib, sorafenib, pazopanib, imatinib, and vemurafenib) for a variety of diseases. Only in the case of RCC patients treated with sunitinib (*n* = 147) did the authors find a correlation between the development of macrocytosis and MCV increases >10 fL, and total survival time (HR = 0.61, *p* = 0.031 and HR = 0.58, *p* = 0.016, respectively).

The mechanism whereby sunitinib causes MCV elevation has not yet been definitively established. Potential causes should include therapy-induced hypothyroidism, folic acid and cobalamin deficiencies, and sunitinib’s inhibition of c-Kit kinase [11].

The earliest available information about sunitinib therapy and MCV elevation was a study published by Gillesenet et al. [17] in the New England Journal of Medicine. They described the development of macrocytosis in 6 mRCC patients treated with sunitinib. The initial treatment dosage was 37.5 mg/day, administered on a continuous schedule. Macrocytosis was observed after 3–4 months of treatment. Cobalamin deficiency, along with proper folic acid plasma concentrations, was found in 5 patients. Five patients had euthyreosis, and one had subclinical hypothyroidism. No anemia was found. Patients with cobalamin deficiency received supplementation intramuscularly. Authors concluded that both macrocytosis and cobalamin deficiency could be caused by sunitinib-induced inhibition of cobalamine absorption in the digestive track. We should note, however, that this study described only patients who developed macrocytosis, and cobalamin levels were not compared across patients with and without macrocytosis. Billemont et al. [18] came to a similar conclusion; they observed MCV elevation in all 40 of their mRCC patients, treated with sunitinib (initial dosage of 50 mg/day on a 6-week schedule: 4 weeks on, 2 weeks off). They found lower levels of cobalamine and folic acid in patients with macrocytosis (MCV > 100 fL) than in those without. They did not find a relationship between these hematological disturbances and thyroid dysfunction.

Rini et al. [19] carried out a detailed analysis of hematological parameters in patients treated with sunitinib. They found MCV elevation, with accompanying but nonsignificant lowered HGB concentrations; MCV elevation was greater in patients with biochemical characteristics of hypothyroidism, but the development of macrocytosis was not limited solely to this group. Patients who had anemia were not found to have reticulocytosis, which could explain MCV elevation (since statistically significant rise in the number of reticulocytes causes the elevation of overall MCV). In trepanobiopsy, the marrow of patients treated with sunitinib was hypocellular, with (reduced) trilineage hematopoiesis, without the dominance of any one lineage. This could indicate the inhibition of marrow function at the stem cell level, which could be a consequence of sunitinib’s activity on c-Kit kinase. This hypothesis is further supported by observations of gastrointestinal stromal tumor (GIST) patients treated with imatinib, a c-Kit inhibitor, 42 % of whom exhibited macrocytosis [20]. On the other hand, mRCC patients treated with sorafenib, whose activity on c-Kit is low, were not found to have macrocytosis. In another study, which evaluated macrocytosis in 29 patients treated with TKIs for various illnesses, MCV elevation was found only in mRCC and breast cancer patients receiving sunitinib and GIST patients receiving imatinib. Other drugs, i.e., sorafenib, erlotinib, and B12992, were not found to affect MCV values [11].

## Conclusion

Our results might suggest that the development of macrocytosis could be a pharmacodynamic marker of sunitinib’s activity in the body and a predictive PFS marker. MCV increases that appear as early as after the first treatment cycle could also have significant predictive value, enabling early evaluation of the drug’s activity. Our small sample size, however, is a major limitation of our study, and our findings must therefore be tested in larger studies in the future.
